# Immunological response to COVID-19 and its role as a predisposing factor in invasive aspergillosis

**DOI:** 10.18502/cmm.6.4.5442

**Published:** 2020-12

**Authors:** Mahin Tavakoli, Tahereh Shokohi, Cornelia Lass Flörl, Mohammad Taghi Hedayati, Martin Hoenigl

**Affiliations:** 1 Invasive Fungi Center, Communicable Diseases Research Institute, Mazandaran University of Medical Sciences, Sari, Iran; 2 Department of Medical Parasitology and Mycology, School of Medicine, Babol University of Medical Sciences, Babol, Iran; 3 Department of Medical Mycology, School of Medicine, Mazandaran University of Medical Sciences, Sari, Iran; 4 nstitute of Hygiene and Medical Microbiology, Medical University of Innsbruck, Innsbruck, Austria; 5 Division of Infectious Diseases and Global Health, University of California San Diego, La Jolla, California, USA; 6 Section of Infectious Diseases and Tropical Medicine, Medical University of Graz, Graz, Austria

**Keywords:** Aspergillosis, *Aspergillus fumigatus*, Immune responses, SARS-CoV-2

## Abstract

The world is involved with a pandemic coronavirus disease 2019 (COVID-19) caused by severe acute respiratory syndrome coronavirus 2. The clinical manifestations of reported COVID-19-associated pulmonary impairments range from asymptomatic infections to a pneumonia-induced acute respiratory distress syndrome that requires mechanical ventilation. Fungal superinfections complicating the clinical course remain underexplored. Angiotensin-converting enzyme 2, the receptor for COVID-19 that is mainly expressed in airway epithelia and lung parenchyma, is considered an important regulator of innate immunity. With regard to the viral-cell interaction, imbalanced immune regulation between protective and altered responses caused by the exacerbation of inflammatory responses should be considered a major contributor to secondary pulmonary aspergillosis. In addition, the complex inherited factors, age-related changes, and lifestyle may also affect immune responses. The complication and persistence of invasive aspergillosis have been well described in patients with severe influenza or COVID-19. However, there is a scarcity of information about the immunological mechanisms predisposing patients with COVID-19 to fungal co-infections. Therefore, this study was conducted to investigate the aforementioned domain.

## Introduction

The world is in the middle of a pandemic viral disease, coronavirus disease 2019 (COVID-19) caused by severe acute respiratory syndrome coronavirus 2 (SARS-CoV-2) ( 1); however, fungal superinfections complicating the clinical course remain underexplored. The clinical course of COVID-19 can be divided into three phases, namely 1) Phase I, referring to the asymptomatic latent period from the increase of viral load to detectable and transmissible levels, 2) Phase II, referring to non-severe symptomatic infection which is the time of viral shedding and clinical symptoms presentation, and 3) Phase III, referring to severe respiratory infection leading to acute respiratory distress syndrome (ARDS) ( 1). The last phase is characterized by high viral load, immunopathological damage, and hypoxemia, as well as the radiological progression of pneumonia that can even lead to the development of ARDS and the use of mechanical ventilation. 

Since fungal superinfections may develop in the middle and late phases of COVID-19, careful attention should be directed to the use of empirical treatments of antifungal superinfections and the need for appropriate and timely clinical follow-ups for the post-viral infectious complications ( 1). Invasive pulmonary aspergillosis (IPA) is a well-known complication acquired mainly through the inhalation of airborne conidia of *Aspergillus* species, particularly *Aspergillus fumigatus*, and is responsible for considerable mortality in immunocompromised and/or neutropenic patients ( 1). 

The complication and persistence of IPA have been well described in patients with or without classic risk factors, including patients with severe influenza ( 1) or COVID-19 ( 2). Nevertheless, little is known about immunological mechanisms predisposing patients with severe viral infections to fungal co-infections. 

## Immune responses induced by COVID-19 infection

Severe acute respiratory syndrome coronavirus 2, sharing many similarities with SARS-CoV, predominantly affects the respiratory tract through a specific receptor, namely angiotensin-converting enzyme 2 (ACE2) ( 1). This receptor is primarily found in the epithelial cells of the intestine, kidney, and lower respiratory tract rather than the upper airway ( 1). Moreover, this enzyme (i.e., ACE2) is an important regulator of innate immunity (with the expression of antimicrobial peptides) and gut microbial ecology ( 3). Although the ubiquitously expressed interferons (IFN)-α/β are believed to be dominant antiviral pathways repressing SARS‐CoV infections, the virus remains highly pathogenic. Upon the virus-cell interaction, despite the anti-inflammatory effects of IFN-λ, type I IFNs (i.e., IFN-α/β) trigger a robust pro-inflammatory response. Hyper-production of mainly pro-inflammatory cytokines, such as interleukin (IL)-1β, IL-6, IL-17A, and tumor necrosis factor alpha, leads to lung injury and aggravates the disease ( 4). 

## Relationship between immune responses induced by SARS-CoV-2 and invasive aspergillosis

During severe COVID-19, the release of damage-associated molecular patterns may aberrantly stimulate toll-like receptors that exacerbate the inflammatory responses and favor COVID-19-associated IPA (CAPA) ( 5, 6). Increased production of IL-6 by epithelial cells is also found following infection with *A. fumigatus*; therefore, the IL-6 driven cytokine storm during the co-infection may lead to the apoptosis of epithelial cells, and subsequently severe ARDS or even death ( 6). In such circumstances, most antifungal therapy failures may be attributed to the patient's previous exposure to antifungals. 

However, in some patients with severe COVID-19 infection, co-infection with triazole-resistant *A. fumigatus* is associated with environmental triazole fungicide use and inhalation of resistant *A. fumigatus* conidia ( 7, 8). Additionally, fungal sensing mediated by the binding of IL-17A to swollen conidia in *A. fumigatus*, rather than resting conidia, may result in the enhancement of biofilm formation, which increases its persistence and antifungal resistance ( 5). Nonetheless, IFN-λ produced by intestinal epithelial cells was insufficient to restrict virus-induced damage within epithelial cells in mice. Consequently, the maximum production of IL-22 by innate lymphoid cells at mucosal surfaces is also needed for the optimal activation of signal transducer and activator of transcription 1, resulting in the effective induction of IFN-stimulated genes (ISGs) for improving antiviral responses ( 4). Typically, interferon-inducible transmembrane proteins (IFITM), as the critical ISG products, are believed to prevent efficient virus-cell fusion ( 6). 

It is suggested that the clinical use of amphotericin B (AMB) for the treatment of serious fungal infections can enhance the infectious entry of SARS-CoVs by evading the IFITM3-mediated restriction ( 9 ). Nevertheless, itraconazole and posaconazole inhibited influenza virus in vitro and in vivo through both IFN-mediated antiviral responses and induced imbalance of cellular cholesterol. Therefore, these antifungals are considered to be the potential antiviral candidates against COVID-19 infection; however, further investigations are required in this regard ( 10), highlighting the caution in the use of AMB for the treatment of COVID-19 with systemic fungal infections. 

According to available evidence of bats carrying CoVs, the presence of fungus may increase shedding and replication of the latent virus that was normally suppressed by the host in intestinal epithelial cells in order to establish subsequent infection ( 11). In this regard, the secondary infection reduces innate anti-viral response through the suppression of mitogen-activated protein kinase-related genes. A high expression level of anti-inflammatory cytokine IL-10 and lower levels of suppressor of cytokine signaling 6 transcripts, reduced IL-22 signaling, required for induction of ISGs expression, and neutrophil infiltration of the lung interstitium. Notably, co-infected bats showed a significantly higher level of immunoglobulin G compared with that in bats infected only with CoV. The upregulation of the renin-angiotensin system, accomplished with ACE2, was found to seemingly affect the rate of cell proliferation in the intestines, as well as being a potent biomarker for the severity of pulmonary and intestinal damages. 

The use of systemic and inhaled corticosteroids, systemic and inhaled antibiotics even for a short time, and supplemental oxygen, in addition to viral exposure, would contribute to the promotion of a shift in the composition and behavior of the local microbial community with potentially pathogenic fungi that elicit further niche inflammation ( 12). In this case, patients are predisposed to colonization with either commensal fungi or exogenous mold opportunistic pathogens, such as *A. fumigatus*, that often tend to develop influenza-associated IPA; the condition that leads to a high mortality rate, especially in critically ill patients ( 13). Moreover, bacterial derived pathogen-associated molecular patterns, including peptidoglycans, lipopolysaccharide, and lipoteichoic acid, apparently behaves in a synergistic manner to significantly increase the production of gliotoxin (GT) by *A. fumigatus*. Not only GT suppresses the immune response but also it exhibits both a general anti-biofilm effect on different bacteria and an antiviral activity against the influenza A virus ( 14). 

The use of corticosteroids suppresses the production and function of IFNs by the induction of suppressor of cytokine signaling 1 or epigenetic alterations and consequently downregulates IFN-λ gene expression and translation ( 15). On the other hand, this drug tends to favor Th2 immune responses leading to increased production of IL-10 that result in the suppression of innate immune responses and an increase in viral shedding ( 11, 15). The adverse effects of long-term and continual administration of high doses of corticosteroids for SARS infections treatment not only upregulated the ACE2 receptor but also influenced the tropism of *A. fumigatus* for different organs to develop disseminated and fatal aspergillosis ( 16). 

A total of 29% of cases with CAPA had previously received systemic corticosteroids, thus far ( 6). The interactions between bronchial epithelial cells and *A. fumigatus* conidia may activate the ACE2 transcription factor leading to cell wall degradation; therefore, the use of corticosteroids can influence the virulence of *A. fumigatus*. Helioswilton et al. ( 17) reported an increasing trend in lung fungal burden, mortality, and pulmonary inflammatory responses in non-neutropenic mice that were immunosuppressed with cortisone acetate and infected with the *∆ace2* mutant strain of *A. fumigatus*. Although there is insufficient data on ligands of *A. fumigatus* conidia involved in the interaction with epithelial cells, it is suggested that conidial adherence can be mediated by both anchorage receptors and fucose-specific lectin receptor to provoke fungistatic activity which depends on phosphoinositide 3 (PI3)-kinase signaling pathway ( 18). The inhibition of this pathway may increase the germination of extracellular conidia; however, internalized conidia do not germinate in A549 cells ( 18). 

The main question here seems to be whether the ACE2 receptor plays a pivotal role in either binding or increasing germination of the extracellular conidia in order to enhance the capability of *A. fumigatus* for infection. Theoretically, corticosteroids can inhibit the PI3 signaling pathway and increase the germination of the extracellular conidia attached to the surface of bronchial epithelial cells ( 19). The inhibition of neutrophil and monocyte activities may result in anti-inflammatory reactions and B- and T- cell-mediated immune suppression in patients receiving steroids. Furthermore, it can accelerate the development of invasive aspergillosis via the shift from pulmonary colonization to invasion ( 12). Monocytes may contribute to thrombosis and local lung tissue injury during *A. fumigatus* infection, leading to the overexpression of urokinase-type plasminogen activator, urokinase-type plasminogen activator receptor, plasminogen activator inhibitor, pentraxin-3, and intercellular adhesion molecule-1 ( 20, 21). 

**Figure 1:**
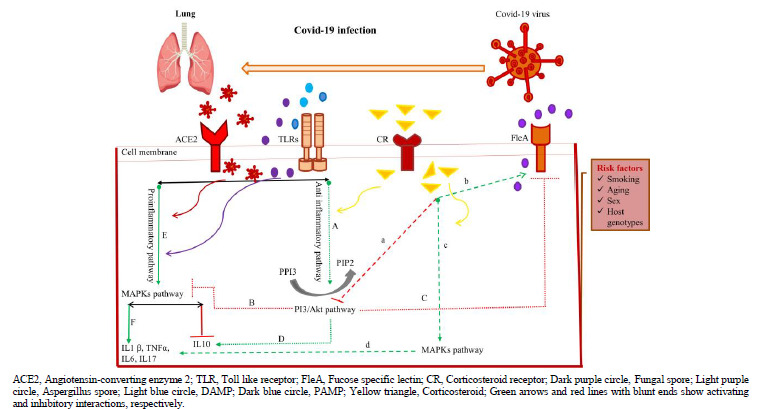
Schematic diagram of PI3K and MAPK cell signaling pathways. Cross-talk of PI3K and MAPK pathways influence eventually each other to maintain organ functions through moderate expression of proinflammatory cytokines such as IL-1 ß, IL-6, IL17, and TNF-α. During COVID-19 infection, ACE2 receptor interaction may induce the receptor signaling. Subsequently, an adaptive immune response initiates through the PI3K, thereby increasing MAPK pathway activity and leading to proinflammatory cytokines production. ACE2 activation can also induce TLR signaling upon interaction with PAMPS, DAMPs, and fungal pathogens resulting in the exaggerated production of proinflammatory cytokines (E and F). Across cell membranes, low-dose corticosteroids bind to CRs and trigger PI3/Akt signals via downstream MAP kinases to suppress inflammation responses (A-D). The steroid binding indeed does interact with FleA, a specific receptor site for *A. fumigatus* and show an inhibitory activity against the entry of this fungus into the cell. Long-term and continual administration of high doses of corticosteroids in return limit PI3/Akt pathway resulting in the upregulation of proinflammatory cytokines but also the robust activation of FleA signaling pathway, thereby facilitating the entry of Aspergillus spores into cells (a-d).

**Figure 2:**
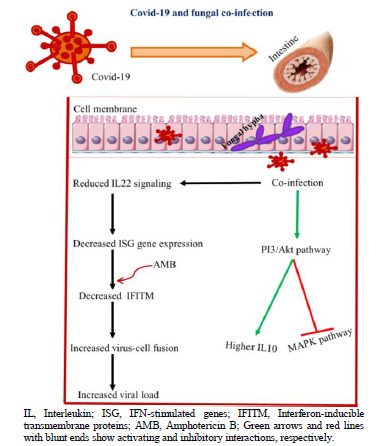
Schematic diagram representative a viral-fungal co-infection. this Coinfection can trigger cross activation of both MAPK and PI3 pathways leading to the suppression of proinflammatory cytokines and an increased level of IL10, reduced IL22 signaling, and ISG gene expression. In this pathway, AMB target IFITM, as the critical ISG products and increase fusion of the virus with host cell membrane and viral load.

The factors of age, gender, and lifestyle, such as smoking, not only upregulate ACE2 receptors but also increase microbial adhesions to airway cells. These additional risk factors for severe mixed infections might implicate in the immune responses against influenza infections ( 22 ). Figures 1 and 2 provide the schematic details on these mentioned processes. Host genotype can also influence the possible outcome of a disease. A large body of evidence has been provided regarding the relationship between the genetic variation of major histocompatibility complex and a wide range of infections potentially posing a global health threat, such as SARS ( 23). Accordingly, multiple polymorphic human leukocyte antigen (HLA) alleles are involved in the risk of SARS-CoV and Middle East respiratory syndrome coronavirus diseases, including HLA-DR subtype B1*1202, HLA-B*4601, HLA-B*0703, HLA-Cw*0801, HLA-DRB1*11:01, and HLA-DQB1*02:02 ( 23). Among the aforementioned HLA polymorphisms, HLA-DR subtypes DRB1*1202 and DRB1*1101 are also associated with susceptibility to allergic bronchopulmonary aspergillosis caused by *A. fumigatus* ( 24). 

## Conclusion

It can be concluded that the immune responses caused by SARS-CoV-2 can increase the risk of fungal superinfections in patients with COVID-19 which may lead to an increase in mortality rate. 
